# Improving DirectLiNGAM for high-dimensional microbiome data: roots screening and eBIC based model selection

**DOI:** 10.3389/fsysb.2026.1835323

**Published:** 2026-06-26

**Authors:** Francesco Canonaco, Enzo Acerbi, Fabio Stella

**Affiliations:** 1 Minutia.AI Pte. Ltd., Singapore, Singapore; 2 Department of Informatics, Systems and Communication, University of Milano-Bicocca, Milano, Italy

**Keywords:** causal networks, DirectLiNGAM, extended BIC, microbiome, roots screening

## Abstract

Identifying causal relationships from observational data is a central challenge in gut microbiome research, where complex, multivariate interactions shape host health and disease. These data are typically high-dimensional and sample-limited, creating substantial obstacles for causal discovery and motivating the development of methods tailored to this regime. In this study, we address this challenge by focusing on DirectLiNGAM and introducing two complementary methodological improvements designed to facilitate its practical application in microbiome data. Specifically, we propose two extensions to the DirectLiNGAM algorithm targeting prior knowledge extraction via roots screening and model selection via the integration of the extended BIC criteria. Together, these contributions extend the applicability of DirectLiNGAM to microbiome systems without altering the core modeling assumptions of the method. We validated the proposed methodology through a rich set of numerical experiments on synthetic data and demonstrate its application on a real biological dataset. This work supports the wider adoption of LiNGAM-based approaches for causal discovery in systems biology and related domains.

## 1 Introduction

Understanding the causal relationships that govern complex biological systems remains a central challenge in systems biology (Faust and Raes, 2012). While association-based methods are widely used to characterize relationships among genes, proteins and microbial taxa (Oña et al., 2025; Szklarczyk et al., 2021), they are limited by the fact that association alone does not reveal causal mechanisms (Pearl, 2009).

Among complex biological systems, the human microbiome has emerged as a central research topic in human biology and biomedicine, with the field gradually shifting from association-based analysis to causal assessments (Moreno-Indias et al., 2021).

However, inferring microbial causal interactions from observational data alone remains challenging due to the high dimensionality of taxonomic profiles, compositional constraints, sparsity, absence of ground truth, and limited cohort size, among other limitations (Faust, 2021). Moreover, the theoretical assumptions required by causal models, such as acyclicity and the absence of unmeasured confounders, are rarely perfectly satisfied in microbiome ecosystems. Although these limitations make applying causal models to observational data highly challenging, moving beyond simple associations remains a necessary step for systems biology. For this reason, microbiome science requires causal discovery tools that can scale computationally. A variety of causal discovery approaches have been proposed for reconstructing directed acyclic graphs (DAGs) from observational data, including constraint-based methods, score-based search procedures and structural equation modeling frameworks (Spirtes et al., 2000). Among the latter, the direct linear non-Gaussian acyclic model (DirectLiNGAM) has attracted attention specifically because of its computational efficiency in identifying causal structures under the assumptions of non-Gaussian noise, acyclicity, linearity and no hidden common causes (Shimizu et al., 2011). In contrast to methods that only recover Markov equivalence classes, DirectLiNGAM guarantees the identification of a causal ordering from purely observational data in a finite number of steps, provided that its assumptions hold. Constraint-based algorithms typically return a completed partially directed acyclic graph (CPDAG), which represents the Markov equivalence class of directed acyclic graphs that are observationally indistinguishable based on conditional independence relations. Consequently, multiple DAGs may be compatible with the same data-generating process, leaving the direction of some edges unresolved. In microbiome research, several approaches have been proposed to infer interactions between microbial taxa. Association-based network inference methods are commonly used to identify co-occurrence patterns (Faust et al., 2012), while models such as Granger causality (Mainali et al., 2019; Sugihara et al., 2012) and generalized Lotka-Volterra systems exploit longitudinal data to characterize temporal dependencies between microbial species (Stein et al., 2013). However, these approaches require longitudinal data, which remain relatively scarce in microbiome studies. More recently, DirectLiNGAM has been applied to infer microbial interactions, providing a more robust framework for distinguishing between potential causal drivers and simple association patterns (Miyajima et al., 2024; Koshida et al., 2023; Miyajima et al., 2022).

Despite attractive theoretical properties, the practical application of DirectLiNGAM in high-dimensional biological settings presents some challenges. First, the algorithm requires repeated evaluation of dependence-based scores across candidate variables during the sequential identification of causal ordering, leading to considerable execution time as the number of variables increases. Second, adjacency matrix estimation is based on node-wise model selection procedures that can become less accurate in sample-limited regimes, where the number of observations is only moderately larger than the number of variables. As a consequence, graph density can grow and false positive edges may accumulate when classical information criteria are used without additional sparsity control.

In this work, we address these limitations by proposing two methodological improvements to the DirectLiNGAM framework; *i*) we introduce a *roots screening* strategy designed to improve computational scalability in high-dimensional settings by identifying candidate exogenous variables prior to full recursive ordering, *ii*) we integrate an extended Bayesian Information Criterion (eBIC) into the adjacency matrix estimation stage to enhance model selection reliability in high-dimensional sample-limited regimes. Together, these contributions aim to preserve the identifiability advantages of DirectLiNGAM while improving its practical applicability to high-dimensional biological data.

The paper is organized as follows. Section 2 introduces the proposed methodological improvements to the DirectLiNGAM framework. Section 3 presents the results of the numerical experiments conducted on synthetic data and illustrates how the proposed methodological innovations can be applied to analyze real-world microbiome data in the form of taxonomical abundance profiles. Finally, Sections 4 and 5 discuss the biological implications and our findings and conclude with a summary of the major contributions of the paper.

## 2 Materials and methods

### 2.1 Problem setting and preliminaries

#### 2.1.1 LiNGAM: Linear non-Gaussian acyclic model

We consider the problem of causal discovery under the LiNGAM framework, originally introduced by (Shimizu et al., 2006). We assume that the observed data are generated by a process that can be represented by a DAG encoding direct causal relationships among variables.

The DAG structure is described by an m×m adjacency matrix $B=\left\{b_{ij}\right\}$ , where each coefficient $b_{ij}$ quantifies the direct causal effect of variable $x_{j}$ on variable $x_{i}$ . Let k(i) denote the position of variable $x_{i}$ in an unknown *causal ordering*, such that no variable appearing later in the ordering has a directed path to any earlier variable. Under this ordering, an edge from $x_{j}$ to $x_{i}$ is allowed only if k(j)<k(i) . The relationships between variables are assumed to be linear, and each observed variable $x_{i}$ is assumed to have zero mean. The data-generating process can then be written as

$$x_{i}=\underset{k\left(j\right)<k\left(i\right)}{\sum }b_{ij}x_{j}+e_{i},$$ (1)

where $e_{i}$ are continuous random variables having non-Gaussian distributions with zero mean and non-zero variance, and $e_{i}$ are independent of each other so there are no latent confounding variables. Equation 1 can be rewritten in compact form as in Equation 2:

x=Bx+e, (2)

where x is a *p*-dimensional random vector, **e** is the *p*-dimensional error vector, and B could be permuted by simultaneous equal row and column permutations to be strictly lower triangular due to the acyclicity assumption (Bollen, 1989).

To illustrate this framework, consider a simple three-variable system where the variables follow a linear causal chain: $x_{1}\to x_{2}\to x_{3}$ . The structural equations for this system are given by:

$$\begin{array}{cc} x_{1} & =e_{1}\\ x_{2} & =b_{21}x_{1}+e_{2}\\ x_{3} & =b_{32}x_{2}+e_{3} \end{array}$$ (3)

In the example Equation 3, $x_{1}$ is an *exogenous* variable (a root node) because it is only determined by its external noise term $e_{1}$ . The variables $x_{2}$ and $x_{3}$ are *endogenous*, as they depend on the values of their respective causal parents. The corresponding adjacency matrix B is:

$$B=\left(\begin{array}{ccc} 0 & 0 & 0\\ b_{21} & 0 & 0\\ 0 & b_{32} & 0 \end{array}\right)$$

Note that B is strictly lower triangular, which is consistent with the causal ordering π=(1,2,3) .

The goal is to estimate the adjacency matrix B by observing data x only. Early estimation approaches, such as ICA-LiNGAM (Shimizu et al., 2006), relied on independent component analysis to recover the mixing matrix $A={\left(I-B\right)}^{-1}$ , but suffer several drawbacks, including the absence of guaranteed convergence to the correct solution from poor initialization (Himberg et al., 2004) and the need for non-trivial parameter selection. To address these limitations, (Shimizu et al., 2011) proposed DirectLiNGAM, which directly estimates the causal ordering by sequentially identifying exogenous variables using independence measures.

#### 2.1.2 DirectLiNGAM: overview of the estimation procedure

DirectLiNGAM provides a sequential procedure for estimating the causal ordering k(i)(i=1,…,p) and the adjacency matrix B under the LiNGAM assumptions. The method exploits a key property of linear non-Gaussian acyclic models: exogenous variables are statistically independent of the residuals obtained by regressing other variables on them.

Let U denote the set of variables that have not yet been assigned to a position in the causal ordering. At each step of the algorithm, DirectLiNGAM evaluates, for each candidate variable $x_{i}\in U$ , a dependence-based score $M\left(x_{i},U\right)$ which quantifies the degree of statistical dependence between $x_{i}$ and the residuals of all other variables in U after linear regression. Intuitively, if $x_{i}$ is exogenous, it should exhibit minimal dependence with these residuals. A variable $x_{i}\in U$ that maximizes the score $M\left(x_{i},U\right)$ is estimated to be a variable that may be the first in the causal ordering. Once the root variable is identified, its effect is removed from the remaining variables $x_{i}\in U$ through linear regression, resulting in a reduced system. This procedure is applied recursively until a complete causal ordering is obtained. Given the estimated ordering, the adjacency matrix B is constructed by regressing each variable on its predecessors in the ordering. In practice, model selection criteria are required to determine which regression coefficients are retained, resulting in a sparse estimate of the underlying DAG.

Although DirectLiNGAM provides a theoretically sound and stable procedure for causal discovery, its practical performance can be challenged by the unique statistical characteristics of biological data. Microbiome and biological networks are characterized not only by high dimensionality and limited samples but also by modular and hierarchical organization (Faust and Raes, 2012; Sorrells and Johnson, 2015; Jiang et al., 2026). In these settings, the sequential evaluation of the score $M\left(x_{i},U\right)$ on all candidate variables can become computationally intractable while the model selection may be affected by the limited number of samples. To address this, we introduce two methodological improvements designed for different data regimes. Our first approach exploits the presence of root nodes to reduce the computational complexity of finding a causal ordering and improves the accuracy of structure recovery, while the second employs the eBIC to improve the estimation of the coefficients matrix B in high-dimensional sample-limited regimes.

### 2.2 Methodological contributions

#### 2.2.1 One shot roots detection via roots screening

In high-dimensional settings, such as those found in microbiome research and systems biology, the computational cost of estimating the causal order in DirectLiNGAM becomes substantial. However, the structured nature of these systems, characterized by a hierarchical organization, presents an opportunity to optimize the search process. In this setting, the identification of exogenous variables represents a critical step in the recursive estimation of the causal structure. We propose a specialization of DirectLiNGAM named *Roots Screening*, designed to identify candidate root nodes prior to executing the full procedure. By isolating exogenous variables in advance, the method provides two main advantages: *i*) reduction of the computational time, and *ii*) improvement of the accuracy of the reconstructed model as a consequence of the reduced dimension of the parameter space.

Specifically, let $m_{1},\ldots ,m_{p}$ denote the ascending ordered absolute values $m_{i}=|M\left(X_{i},U\right)|$ with p≥2 . To ensure numerical stability and scale-invariance, we introduce a stability constant, ε , defined as $10^{-10}$ times the smallest non-zero value among these scores. We then compute the logarithmic differences as in Equation 4:

$$\Delta_{i}=\log_{10}\left(m_{i+1}+\varepsilon \right)-\log_{10}\left(m_{i}+\varepsilon \right)$$ (4)

for i=1,…,p−1 . The optimal split point $i^{⋆}$ is identified by finding the largest jump starting from a meaningful value:

$$i^{⋆}=\arg\;\underset{i}{\max}\left\{\Delta_{i}|m_{i}>10\varepsilon \right\}.$$ (5)

The variables corresponding to indices $1,\ldots ,i^{⋆}$ are then selected as the set of candidate root nodes R as indicated by Equation 5.

Algorithm 1 **Roots Screening procedure for candidate exogenous variables.**

**Require:** Set of dependence scores $\left\{M\left(x_{i},U\right)\right\}$ for $x_{i}\in U$ , stability constant ε

**Ensure:** Set of candidate root nodes R

 1: Compute absolute scores $m_{i}=|M\left(x_{i},U\right)|$

 2: Sort scores in ascending order: $m_{1}\leq m_{2}\leq \cdots \leq m_{p}$

 3: **if** ε is not provided **then**
 4: $\varepsilon \leftarrow \min\left(m_{i}|m_{i}>0\right)\times 1e-10$

 5: **end** **if**
 6: **for** i=1 **to** p−1 **do**
 7: $\Delta_{i}\leftarrow \log_{10}\left(m_{i+1}+\varepsilon \right)-\log_{10}\left(m_{i}+\varepsilon \right)$

 8: **end** **for**
 9: Identify the largest jump starting from a meaningful value:
 10: $i^{⋆}\leftarrow \arg\max_{i}\left\{\Delta_{i}|m_{i}>10\varepsilon \right\}$

 11: $R\leftarrow \left\{x_{j}\text{ corresponding to }m_{1},\ldots ,m_{i^{⋆}}\right\}$

 12: **return** R

It is worth mentioning that the separation criterion in Algorithm 1 is not unique. Alternative strategies could include fixed thresholds, percentile-based selection, or more sophisticated change-point detection procedures Killick et al. (2012). The proposed procedure is motivated by its simplicity and by the expected behavior of $m_{i}=|M\left(X_{i},U\right)|$ , where exogenous variables typically produce substantially smaller values than non-exogenous ones. Moreover, our procedure introduces negligible overhead compared to the recursive estimation required by DirectLiNGAM.

#### 2.2.2 Extended BIC for model selection in DirectLiNGAM

After estimating the causal ordering k(i)(i=1,…,p) , DirectLiNGAM determines the parent set of each node by applying adaptive LASSO regression restricted to its predecessors in the ordering. In the standard implementation, the optimal support along the regularization path is selected using the Bayesian Information Criterion (BIC) (Neath and Cavanaugh, 2012). In high-dimensional and sample-limited regimes, the BIC penalty may be insufficient to account for model complexity. In particular, when the number of candidate parents is large, BIC may favor overly dense parent sets, potentially leading to inflated graph connectivity. To improve sparsity control, we replace the BIC-based selection step with an Extended Bayesian Information Criterion (eBIC) (Chen and Chen, 2008), evaluated along the adaptive-LASSO path.

For a candidate parent set of size $c_{i}$ of the variable $x_{i}$ with residual sum of squares RSS , the eBIC score is defined as

eBICi=n⁡log RSSin+ci⁡log(n)+2γeBIC⁡log plocici,

where n denotes the sample size, $p_{{\text{loc}}_{i}}$ is the number of candidate parents for the current node $x_{i}$ , and $\gamma_{\text{eBIC}}\in \left[0,1\right]$ controls the strength of the additional sparsity penalty. The binomial coefficient accounts for the size of the local model space and increases penalization as the number of possible parent subsets grows. When $\gamma_{\text{eBIC}}=0$ , the criterion reduces to the classical BIC. Positive values of $\gamma_{\text{eBIC}}$ encourage sparser parent sets, which is desirable in high-dimensional causal graph selection. In our framework, we set $\gamma_{\text{eBIC}}=0.5$ . This value was established by (Foygel and Drton, 2010) as an optimal and widely adopted default for high-dimensional network inference, providing a theoretical balance between false-positive control and statistical power. Furthermore, selecting this moderate penalty maintains methodological comparability with the baseline DirectLiNGAM, which utilizes adaptive LASSO regression without targeted sparsity tuning.

Notably, this modification affects only the model selection stage and does not alter the estimation of the causal ordering provided by DirectLiNGAM. The assumptions and identifiability of the underlying model remain unchanged.

### 2.3 Experimental setup

In this section, we describe numerical experiments on both synthetically generated data and real world data.

#### 2.3.1 Synthetic data generation and evaluation metrics

To evaluate the scalability of Roots Screening in high-dimensional regimes, we generated synthetic datasets from Barabási-Albert (BA) (Barabási and Albert, 1999) networks. In this model, each newly added node connects to m existing nodes, where m controls the connectivity of the resulting network and therefore the overall graph sparsity. Experiments were conducted by varying the number of variables p∈{100,300,500} , the number of samples n∈{2,000,6,000,10,000} , and the connectivity parameter m∈{1,2,3} . For each parameters configuration, a ground-truth network was generated, synthetic observations were sampled from the corresponding structural equation model, and the causal structure was estimated. Each parameter configuration was repeated 20 times using different random seeds, resulting in different randomly generated networks and datasets for the same parameters setting.

To evaluate model selection performance in sample-limited regimes ground-truth DAGs were created by controlling the number of variables p , the sample size n , and the graph sparsity level as in (Shimizu et al., 2011). The number of variables varied as p∈{50,100,150} . For each value of p , the ground-truth DAG was generated by fixing the number of expected edges for each node as d∈{2,3,4} . The sample size was determined as a multiple of the dimensionality according to n=r⋅p , where r∈{1.5,2.0,3.0,4.0} . These configurations explicitly explore sample-limited regimes in which the number of observations is only moderately larger than the number of variables. For each parameter configuration (p,d,r) , a ground-truth DAG was generated, synthetic observations were sampled from the corresponding structural equation model, and the causal structure was estimated using DirectLiNGAM with both the standard BIC and the proposed extended BIC (eBIC) selection criterion. Each configuration was repeated 100 times using different random seeds to account for variability in the generated graphs and sampled datasets. For both methodologies, structural recovery was evaluated using Structural Hamming Distance (SHD) (Tsamardinos et al., 2006) that represents the total number of edge operations, specifically additions, deletions, and orientation reversals required to transform the estimated DAG into the ground-truth graph, while edge-level performance was assessed using Precision, Recall, F1 score, and mean squared error (MSE).

#### 2.3.2 Curated metagenomic data processing

We used human gut microbiome shotgun metagenomic profiles from the curatedMetagenomicData resource (accessed 2022-11–18), which provides uniformly processed taxonomic profiles and manually curated metadata (Pasolli et al., 2017).

In the curatedMetagenomicData, raw sequencing data are processed through standardized bioinformatics pipelines to generate microbial taxonomic and functional profiles, which are distributed as (Tree)SummarizedExperiment objects with consistent metadata fields. For our analysis, only taxonomic profiles were used and the analysis was restricted to *control* subjects only (defined as *healthy*), selecting stool samples from adults as defined by age_category = adult, and retaining only samples with non-missing age information in the range 18≤age≤107 . These selection criteria were chosen to define a healthy reference cohort with minimized biological heterogeneity.

Starting from the selected stool samples, we applied a series of preprocessing steps described as follows. Species-level abundance profiles were extracted from curatedMetagenomicData using count data as the initial data representation. To ensure adequate sequencing depth, we excluded samples with fewer than one million reads (Gweon et al., 2019). In addition, samples with a median read length below 90 bp were removed (Peabody et al., 2015). To reduce the impact of data sparsity, we retained only species present in at least 30% of the samples (Nearing et al., 2022). Furthermore, species with a mean relative abundance below $1\times 10^{-4}$ were also excluded. The resulting dataset comprised 2,651 samples and 100 species. In order to ensure interpretability and address compositionality after the network estimation, the centered log ratio transformation was applied (Quinn et al., 2019; Gloor et al., 2017).

## 3 Results

### 3.1 Performance on synthetic data

We evaluate the proposed methodological improvements on synthetic datasets and compare their performance with the baseline DirectLiNGAM approach. The experiments are designed to assess both structural accuracy and computational efficiency across different data regimes.

#### 3.1.1 Roots screening in high-dimensional settings

**FIGURE 1:**
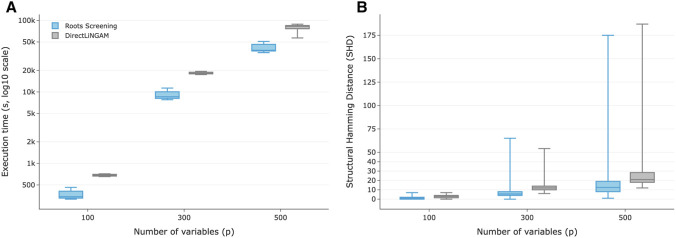
**(A)** Execution time scaling of Roots Screening and DirectLiNGAM in high-dimensional settings. Boxplots show execution time (expressed in seconds, log scale) as a function of the number of variables p for Roots Screening and standard DirectLiNGAM, evaluated on synthetic Barabási–Albert networks with n=6,000 samples. Results are pooled across network connectivity settings (m) . **(B)** Structural recovery performance under the same experimental conditions. Boxplots report the Structural Hamming Distance (SHD) between the estimated and true causal graphs as a function of p , highlighting the improved structural accuracy of Roots Screening in high-dimensional regimes.

Figure 1A reports the execution time (seconds, log scale) of standard DirectLiNGAM and the proposed Roots Screening approach respectively, as a function of p , with results pooled across network connectivity settings. Roots Screening consistently reduces computation time relative to DirectLiNGAM, with increasing gains as dimensionality grows. This highlights the improved scalability in high-dimensional settings of the Roots Screening method. In terms of structural recovery, Roots Screening consistently achieves lower Structural Hamming Distance (SHD) compared to standard DirectLiNGAM in all considered number of variables, as shown in Figure 1B. The improvement becomes more pronounced as the number of variables increases, indicating enhanced robustness of the proposed approach in high-dimensional regimes. While variability in SHD increases with p for both methods, Roots Screening exhibits a lower SHD without affecting Precision, Recall or F1 score as summarized in Table 1, suggesting more stable structure estimation under scale-free network assumptions. Detailed results can be found in the Supplementary Figures S1, S2, S3, S4 and Supplementary Table S1, S2.

**TABLE 1:** Structural and computational performance of Roots Screening and DirectLiNGAM at fixed sample size (n=6,000) . Results are reported as mean ± standard deviation over 20 runs and pooled across network connectivity settings (m) .

p	Method	SHD	MSE $\left(\times 10^{-5}\right)$	Precision	Recall	F1	Exec. Time (s)
100	DirectLiNGAM	2.7±1.5	0.29±0.09	0.984±0.011	1.000±0.000	0.992	683±20
100	Roots screening	1.6±1.5	0.27±0.09	0.991±0.010	1.000±0.000	**0.996**	362±44
300	DirectLiNGAM	12.1±6.2	0.53±2.92	0.976±0.014	1.000±0.001	0.988	18,360±559
300	Roots screening	7.7±10.1	2.64±16.62	0.987±0.014	1.000±0.001	**0.993**	9,019±1,104
500	DirectLiNGAM	29.5±31.9	1.57±7.33	0.971±0.019	1.000±0.001	0.985	80,096±7,432
500	Roots screening	18.1±23.5	0.99±5.48	0.983±0.014	1.000±0.001	**0.992**	40,927±4,816

Bold values indicate the best performance.

#### 3.1.2 Extended BIC in sample-limited regimes

**FIGURE 2:**
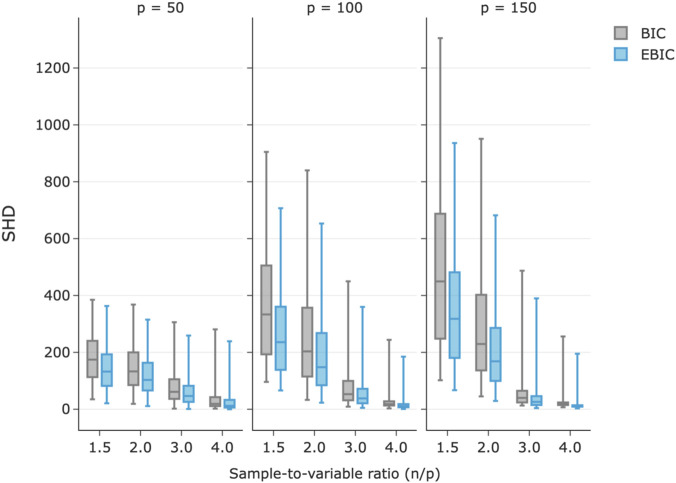
Structural recovery performance of DirectLiNGAM with standard BIC and Extended BIC. Structural Hamming Distance (SHD) is shown as a function of the sample-to-variable ratio $r=\frac{n}{p}$ for increasing dimensionality (p) . Results are pooled across sparsity levels. Boxes indicate interquartile ranges, with medians shown as horizontal lines. Lower values indicate better structural recovery.

As shown in Figure 2, extended BIC consistently improves structural recovery in sample-limited regimes, leading to more accurate graph estimation compared to standard BIC. Results reported in Table 2 confirm this trend, showing higher Precision and F1 score under extended BIC, while Recall and MSE remain largely unchanged. These findings indicate that the extended penalty primarily reduces false positive edges without compromising sensitivity.

**TABLE 2:** Performance comparison between BIC and extended BIC (EBIC) across different sample-to-variable ratios (n/p) for synthetic datasets with p=100 . Results are reported as mean ± standard deviation over repeated runs and are pooled across different number of expected edges per node d∈{2,3,4} .

n/p	Method	SHD	MSE	Precision	Recall	F1
1.5	BIC	365.840±189.456	0.014±0.008	0.283±0.074	0.735±0.061	0.405±0.084
1.5	EBIC	262.160±140.208	0.013±0.007	0.366±0.091	0.724±0.065	0.483±0.093
2.0	BIC	256.150±170.806	0.010±0.008	0.407±0.125	0.835±0.064	0.541±0.125
2.0	EBIC	190.473±129.999	0.010±0.008	0.488±0.135	0.828±0.068	0.608±0.125
3.0	BIC	78.483±72.695	0.003±0.003	0.709±0.136	0.961±0.037	0.811±0.108
3.0	EBIC	57.507±58.171	0.003±0.003	0.774±0.132	0.960±0.038	0.853±0.100
4.0	BIC	28.080±32.206	0.001±0.001	0.863±0.086	0.993±0.014	0.921±0.059
4.0	EBIC	18.257±25.710	0.001±0.001	0.909±0.078	0.993±0.014	0.947±0.052

Bold values indicate the best performance.

### 3.2 Application to biological data

**FIGURE 3:**
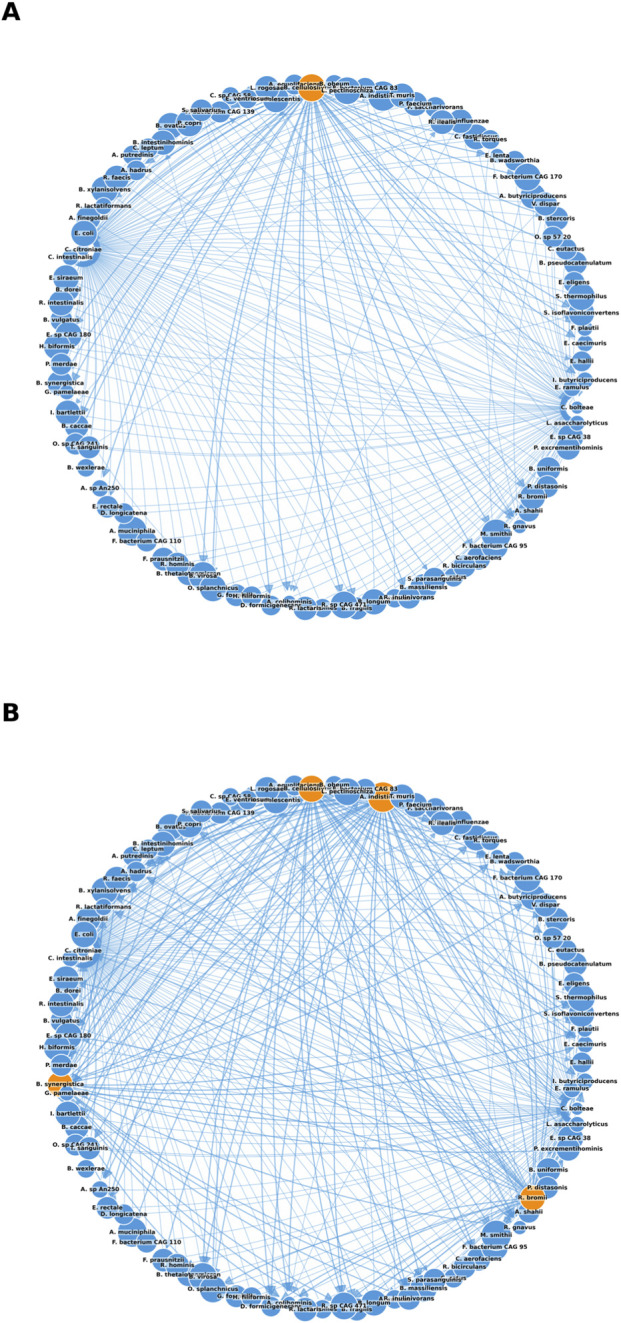
**(A)** Causal network estimated using standard DirectLiNGAM **(B)** Causal network estimated using the proposed Roots Screening specialization of DirectLiNGAM.

Figure 3B illustrates the estimated directed networks obtained with the Roots Screening procedure, while Figure 3A shows the one estimated with the standard DirectLiNGAM. Node size is proportional to out-degree. Orange nodes denote variables identified as root nodes in the estimated causal ordering, while blue nodes represent non-root variables. Using the Roots Screening procedure, multiple taxa were identified as candidate root nodes, namely,: *Bacteroides cellulosilyticus*, *Ruminococcus bromii*, *Alistipes indistinctus*, and *Butyricimonas synergistica*. High-resolution subnetwork visualizations detailing the localized downstream causal footprints of these individual drivers are provided in the (Supplementary Figure S6). For comparison, the corresponding localized neighborhood generated via the baseline framework is reported in Supplementary Figure S5. Interestingly, several of these taxa belong to genera previously discussed in large-scale community representations of the human gut microbiome, which suggests that the inferred structure is at least compatible with broad, previously described microbiome patterns. In particular, the detection of *Bacteroides* associated taxa as roots (*B. cellulosilyticus*) aligns with the corresponding well-known *Bacteroides* enterotype, one of the three enterotypes describing the human gut microbiome (Arumugam et al., 2011). Similarly, the presence of Firmicutes-related taxa such as *R. bromii* as a root is also a match with another enterotype, specifically the Firmicutes one. These correspondence between hubs and enterotypes can be interpreted as alignment of the inferred networks with major ecological functions (e.g., carbohydrate-driven *versus* alternative metabolic regimes) in the human gut microbiome.

In addition, the identification of *Alistipes indistinctus* as a root node may reflect its strong link with host-level environmental factors. Studies show that Alistipes species are linked to protein-rich dietary patterns and bile acid metabolism. These host-driven conditions are known to influence multiple microbial groups simultaneously, which may contribute to its upstream placement in the model-implied ordering.

In contrast, standard DirectLiNGAM identified a single root node, *Bacteroides cellulosilyticus*, resulting in a more centralized upstream structure that is harder to interpret as a direct species-level dependency pattern. In summary, the identification of multiple upstream drivers under Roots Screening is consistent with the expectation that gut microbial ecosystems exhibit modular and partially independent functional hubs rather than a strictly single-root hierarchy.

To demonstrate the application of the eBIC approach on real data and compare it with standard DirectLiNGAM, we uniformly at random sampled a subset of 300 observations, while retaining all 100 species variables.

**FIGURE 4:**
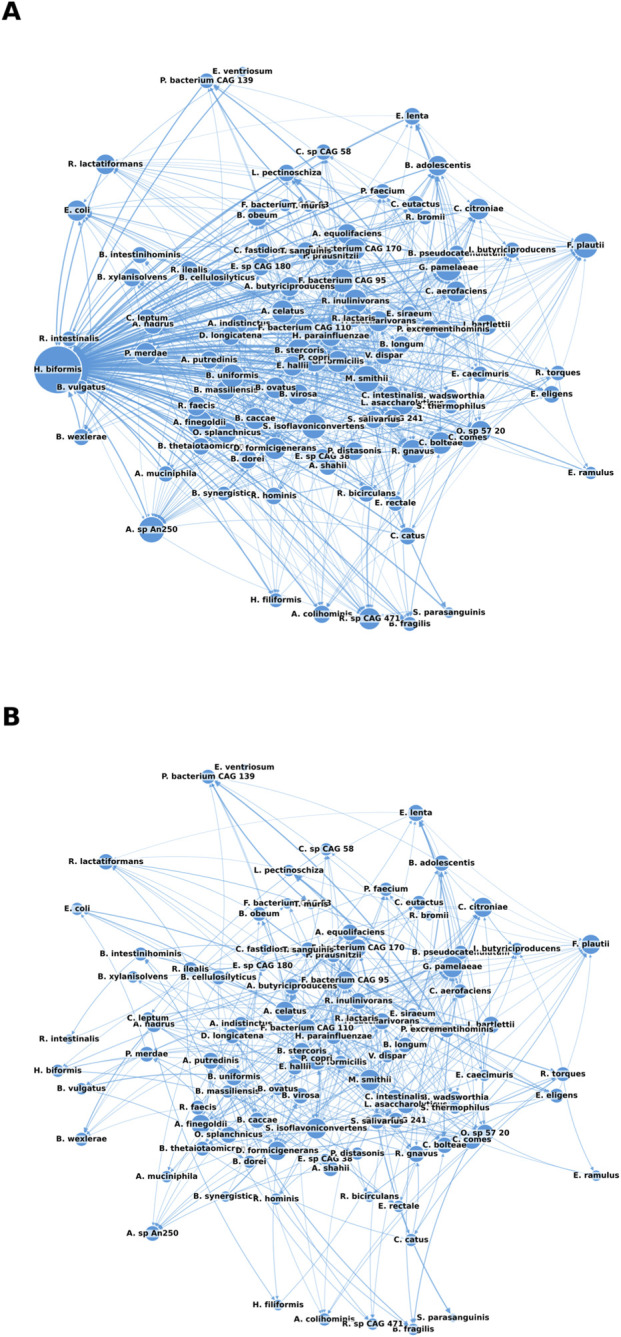
**(A)** Causal network estimated using standard DirectLiNGAM **(B)** Causal network estimated using eBIC.

A qualitative inspection of network hubs highlights additional and substantial differences between the two inferred structures. An analysis of the degree distribution (Supplementary Figure S8) clearly highlights this structural divergence. As mentioned, the network obtained using the proposed eBIC-regularized approach (Figure 4B) exhibits a distributed hub structure involving multiple taxa with well-established roles in the human gut microbiome. In contrast, the network inferred by standard DirectLiNGAM (Figure 4A) more strongly concentrated around a single highly connected node (*Holdemanella biformis*), which results in a highly centralized topology that is harder to interpret biologically, especially as evidence of direct microbial dependencies. In the eBIC-based network, the highest degree nodes include taxa such as *Prevotella copri*, *Methanobrevibacter smithii*, *Alistipes finegoldii* and *Flavonifractor plautii*. Detailed high-resolution visualizations of the localized neighborhoods surrounding these specific taxa are provided in the Supplementary Material (Supplementary Figure S7).

These organisms are widely recognized as important players in the gut ecosystem. For example, *Prevotella copri* is a key representative of the Prevotella enterotype and is associated with carbohydrate fermentation and dietary fiber metabolism (Betancur-Murillo et al., 2022). Similarly, *Methanobrevibacter smithii* is the dominant archaeal methanogen in the human gut: its hub role is biologically plausible given its documented involvement in cross-feeding networks (Catlett et al., 2022). Other prominent nodes in the eBIC network, such as *Gordonibacter pamelaeae*, *Slackia isoflavoniconvertens* and *Asaccharobacter celatus*, belong to the Eggerthellaceae family and are known to participate in the metabolism of dietary polyphenols and other complex compounds (Rodríguez-Daza et al., 2021). Their central position in the network may reflect metabolic dependencies related to dietary substrate processing. Likewise, taxa such as *Dorea formicigenerans* and *Flavonifractor plautii* are involved in short-chain fatty acid production and aromatic compound metabolism, both of which are fundamental processes within gut microbial communities (Mi et al., 2024). Importantly, these hubs span across multiple phylogenetic groups and metabolic clusters. This distributed hub structure is consistent with the current ecological view of the gut microbiome as a system composed by multiple interacting metabolic guilds rather than by a single dominant regulator. In contrast, the network produced by standard DirectLiNGAM displays *Holdemanella biformis* as single dominating hub, which connects to nearly all other nodes. Although *Holdemanella biformis* is a known intestinal commensal, we are unaware of evidence supporting its interpretation as a global driver controlling a large fraction of intestinal microbial interactions. Since microbiome interaction networks are generally expected to exhibit modularity and multiple functional hubs rather than a single organism exerting dominant influence, our results suggest that the proposed methodological improvements produce estimated network structures that are both quantitatively improved and qualitatively more consistent with known high-level ecological organization of the human gut microbiome. This interpretation refers to the global topology of the inferred networks and should not be taken as validation of individual species-to-species causal effects.

## 4 Discussion

As the microbiome field transitions from association-based methodologies to mechanistic approaches, there is a growing need for causal discovery frameworks tailored to the unique properties of microbiome data. In this study, we addressed this need by proposing two methodological advances to the DirectLiNGAM framework that improve scalability and structural recovery in high-dimensional and sample-limited settings typical of microbiome data. Specifically, the proposed *roots screening* strategy relies on the presence of root nodes in microbial interaction networks to reduce the search space of the estimated causal ordering. This resulted in reduced execution time and improved structural recovery (Figure 1; Table 1). In addition, the adoption of the extended Bayesian Information Criterion (eBIC) improved structural recovery and precision in sample-limited regimes as shown in Figure 2 and Table 2. A fundamental challenge in evaluating causal discovery methods on real microbiome data is that the absence of a ground-truth microbial interaction network makes the validation difficult. Nevertheless, we observed several qualitative patterns suggesting that the proposed methodological improvements produce estimated network topologies that are more compatible with current high-level ecological knowledge of the human gut microbiome.

Despite the encouraging results, several limitations of the proposed approach should be considered. The first limitation comes from the linearity and acyclicity assumptions of the LiNGAM framework, which may not fully capture the complexity of microbial interactions (Coyte et al., 2015). A second limitation concerns the potential presence of unobserved confounders. In microbial ecosystems, interactions between species are often mediated by metabolites that are not directly measured in metagenomic datasets. As a result, an inferred causal relationship between two species might reflect the influence of an unobserved metabolite rather than a direct interaction between them (Sazal et al., 2021). Furthermore, while the logarithmic gap statistic utilized in *roots screening* proved empirically robust across diverse network topologies, it remains a heuristic. Subsequent iterations of this methodology could benefit from investigating formal statistical change-point detection algorithms to provide rigorous probabilistic bounds on the roots selection process. Finally, the evaluation of causal discovery methods in microbiome research is mainly based on simulation studies and indirect biological evidence.

Future work could include the integration of additional *omics* data layers, such as metabolomics or metatranscriptomics, helping account for hidden metabolic mediators and reducing the impact of unobserved confounders. In addition, adopting temporal models would provide a more realistic representation of microbial networks. In particular, longitudinal LiNGAM (Kadowaki et al., 2013) could enable the identification of temporal interactions that address the limitation given by the acyclicity assumption. While linear models offer a computationally robust first order approximation, subsequent research could explore non linear causal discovery frameworks to better capture the complex, higher order dynamics inherent to microbial ecosystems.

## 5 Conclusion

This study highlights the potential of DirectLiNGAM framework to move beyond association-based analysis and toward a mechanistic understanding of microbial ecosystems. By improving the scalability and robustness of the DirectLiNGAM framework in high-dimensional and sample-limited settings, the proposed methodological improvements provide a step toward more reliable causal discovery for microbiome research and systems biology. As microbiome datasets continue to grow in size and complexity, causal discovery frameworks will play an increasingly important role in disentangling the mechanisms underlying microbial community structure and their impact on host health. Although inferred networks should not be interpreted as validated microbial interaction maps without additional experimental evidence, they can provide useful hypotheses about directed dependencies and community-level organization.

### 5.1 Reproducibility

All experiments conducted in this study are fully reproducible. The biological application is based on publicly available human microbiome datasets from the curatedMetagenomicData resource. The data used in this work are available in the data folder in the Supplementary Material. All source code required to reproduce the synthetic experiments and the real data application is available at https://github.com/Minutia-AI/Improving-DirectLiNGAM-for-High-Dimensional-Microbiome-Data. The synthetic experiments were implemented to allow parallel execution across multiple cores, enabling efficient evaluation of the different parameter configurations. All experiments were conducted on a dedicated server equipped with a *64-core AMD EPYC 7R32* processor and *124 GiB* of RAM. The host system operated on *Ubuntu 24.04.2 LTS*.

## Data Availability

The original contributions presented in the study are included in the article/Supplementary Material, further inquiries can be directed to the corresponding author.
