# Correction: Improving DirectLiNGAM for high-dimensional microbiome data: roots screening and eBIC based model selection

**DOI:** 10.3389/fsysb.2026.1929002

**Published:** 2026-07-21

**Authors:** Francesco Canonaco, Enzo Acerbi, Fabio Stella

**Affiliations:** 1 Minutia.AI Pte Ltd., Singapore, Singapore; 2 Department of Informatics, Systems and Communication, University of Milano-Bicocca, Milano, Italy

**Keywords:** causal networks, DirectLiNGAM, extended BIC, microbiome, roots screening

The conflict of interest statement was erroneously given as:

‘Authors FC and EA were employed by Minutia.AI Pte. Ltd.

The remaining author(s) declared that this work was conducted in the absence of any commercial or financial relationships that could be construed as a potential conflict of interest’.

The correct conflict of interest statement is:

‘Author FC is funded by Minutia.AI Pte. Ltd. Author EA has equity and is an advisor to Minutia.AI Pte. Ltd.

The remaining author(s) declared that this work was conducted in the absence of any commercial or financial relationships that could be construed as a potential conflict of interest’.

The original version of this article has been updated.

