# Supplementary material for: Improving DirectLiNGAM for high-dimensional microbiome data: roots screening and eBIC based model selection
Source: Front Syst Biol. 2026 Jun 26;6:1835323. doi: 10.3389/fsysb.2026.1835323 (PMC13349759; doi:10.3389/fsysb.2026.1835323)
Supplement: Supplementary file 1 [file Supplementaryfile1.pdf]

Supplementary Material

1 SUPPLEMENTARY TABLES AND FIGURES

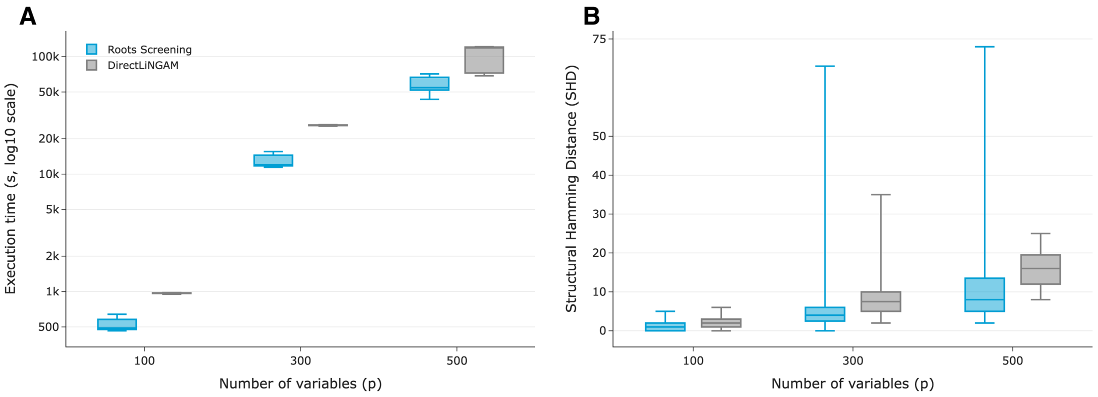

Figure S1. (A) Execution time scaling of Roots Screening and DirectLiNGAM in high-dimensional settings. Boxplots show execution time (expressed in seconds, log scale) as a function of the number of variables p for Roots Screening and standard DirectLiNGAM, evaluated on synthetic Barabási–Albert networks with $n = 10,000$ samples over 20 runs. Results are pooled across network connectivity settings (m). (B) Structural recovery performance under the same experimental conditions. Boxplots report the Structural Hamming Distance (SHD) between the estimated and true causal graphs as a function of p, highlighting the improved structural accuracy of Roots Screening in high-dimensional regimes.

Table S1. Structural and computational performance of Roots Screening and DirectLiNGAM at fixed sample size (n = 10,000). Results are reported as mean ± standard deviation over 20 runs and pooled across network connectivity settings (m).

p	Method	SHD	MSE ($\times 10^{-5}$)	Precision	Recall	F1	Exec. time (s)
100	DirectLiNGAM	2.0 ± 1.4	0.17 ± 0.06	0.988 ± 0.010	1.000 ± 0.000	0.994	968 ± 7
100	Roots Screening	**1.2** ± 1.1	**0.15** ± 0.06	**0.993** ± 0.007	1.000 ± 0.000	**0.996**	**520** ± 58
300	DirectLiNGAM	8.1 ± 4.8	**0.62** ± 3.90	0.984 ± 0.011	1.000 ± 0.001	0.992	26,003 ± 193
300	Roots Screening	**5.5** ± 8.7	2.23 ± 16.40	**0.991** ± 0.013	1.000 ± 0.001	**0.995**	**12,849** ± 1,439
500	DirectLiNGAM	16.3 ± 4.8	**0.06** ± 0.23	0.981 ± 0.010	1.000 ± 0.001	0.990	103,452 ± 23,126
500	Roots Screening	**10.5** ± 11.8	0.54 ± 0.28	**0.990** ± 0.008	1.000 ± 0.001	**0.995**	**57,540** ± 8,020

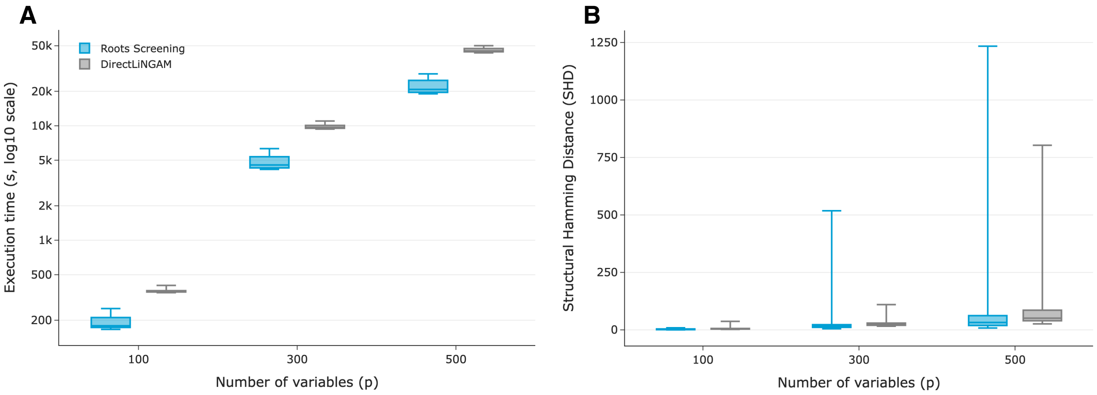

Figure S2. (A) Execution time scaling of Roots Screening and DirectLiNGAM in high-dimensional settings. Boxplots show execution time (expressed in seconds, log scale) as a function of the number of variables p for Roots Screening and standard DirectLiNGAM, evaluated on synthetic Barabási–Albert networks with $n = 2,000$ samples over 20 runs. Results are pooled across network connectivity settings (m). (B) Structural recovery performance under the same experimental conditions. Boxplots report the Structural Hamming Distance (SHD) between the estimated and true causal graphs as a function of p, highlighting the improved structural accuracy of Roots Screening in high-dimensional regimes.

Table S2. Structural and computational performance of Roots Screening and DirectLiNGAM at fixed sample size (n = 2,000). Results are reported as mean $\pm$ standard deviation over 20 runs and pooled across network connectivity settings (m).

p	Method	SHD	MSE ($\times 10^{-5}$)	Precision	Recall	F1	Exec. time (s)
100	DirectLiNGAM	5.5 $\pm$ 4.6	4.26 $\pm$ 25.44	0.969 $\pm$ 0.022	1.000 $\pm$ 0.001	0.984	365 $\pm$ 19
100	Roots Screening	**2.9** $\pm$ 2.3	**0.87** $\pm$ 0.28	**0.983** $\pm$ 0.015	1.000 $\pm$ 0.000	**0.992**	**192** $\pm$ 26
300	DirectLiNGAM	**28.7** $\pm$ 16.3	**2.58** $\pm$ 8.36	0.949 $\pm$ 0.025	**1.000** $\pm$ 0.001	0.974	9,831 $\pm$ 471
300	Roots Screening	31.1 $\pm$ 72.8	49.10 $\pm$ 341.68	**0.959** $\pm$ 0.058	0.999 $\pm$ 0.003	**0.979**	**4,829** $\pm$ 644
500	DirectLiNGAM	91.0 $\pm$ 123.8	**13.35** $\pm$ 46.29	0.926 $\pm$ 0.051	0.999 $\pm$ 0.006	0.961	45,783 $\pm$ 1,898
500	Roots Screening	**81.3** $\pm$ 190.4	16.29 $\pm$ 73.01	**0.947** $\pm$ 0.072	0.999 $\pm$ 0.006	**0.972**	**22,099** $\pm$ 3,006

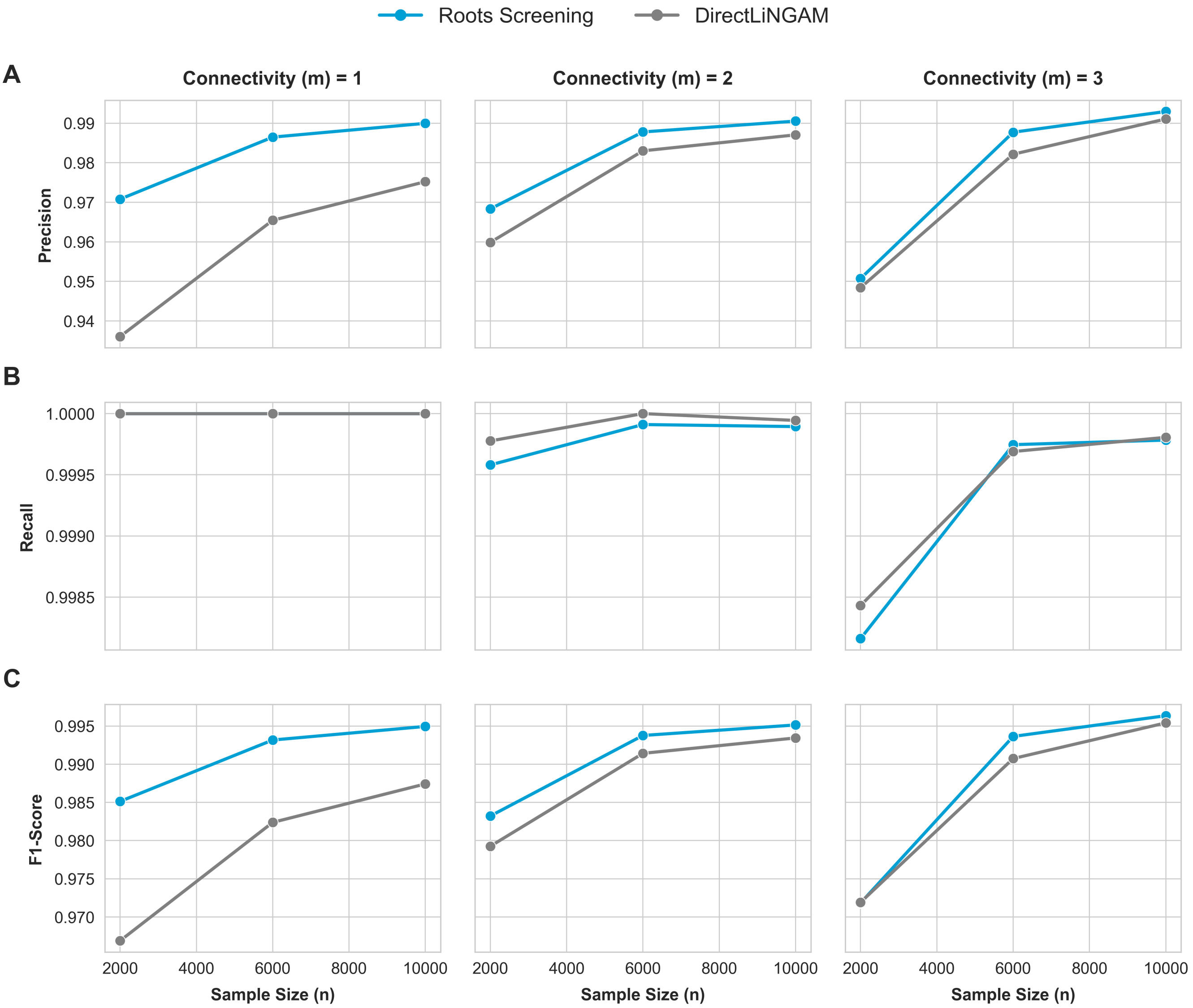

Figure S3. Edge detection performance across varying sample sizes (n) and connectivity regimes (m) averaged over 20 independent runs. Panels evaluate **(A)** Precision, **(B)** Recall, and **(C)** F1-Score. The proposed Roots Screening method (blue) maintains near-optimal performance across all network densities, demonstrating significant robustness to increasing network connectivity compared to the baseline DirectLiNGAM approach (gray).

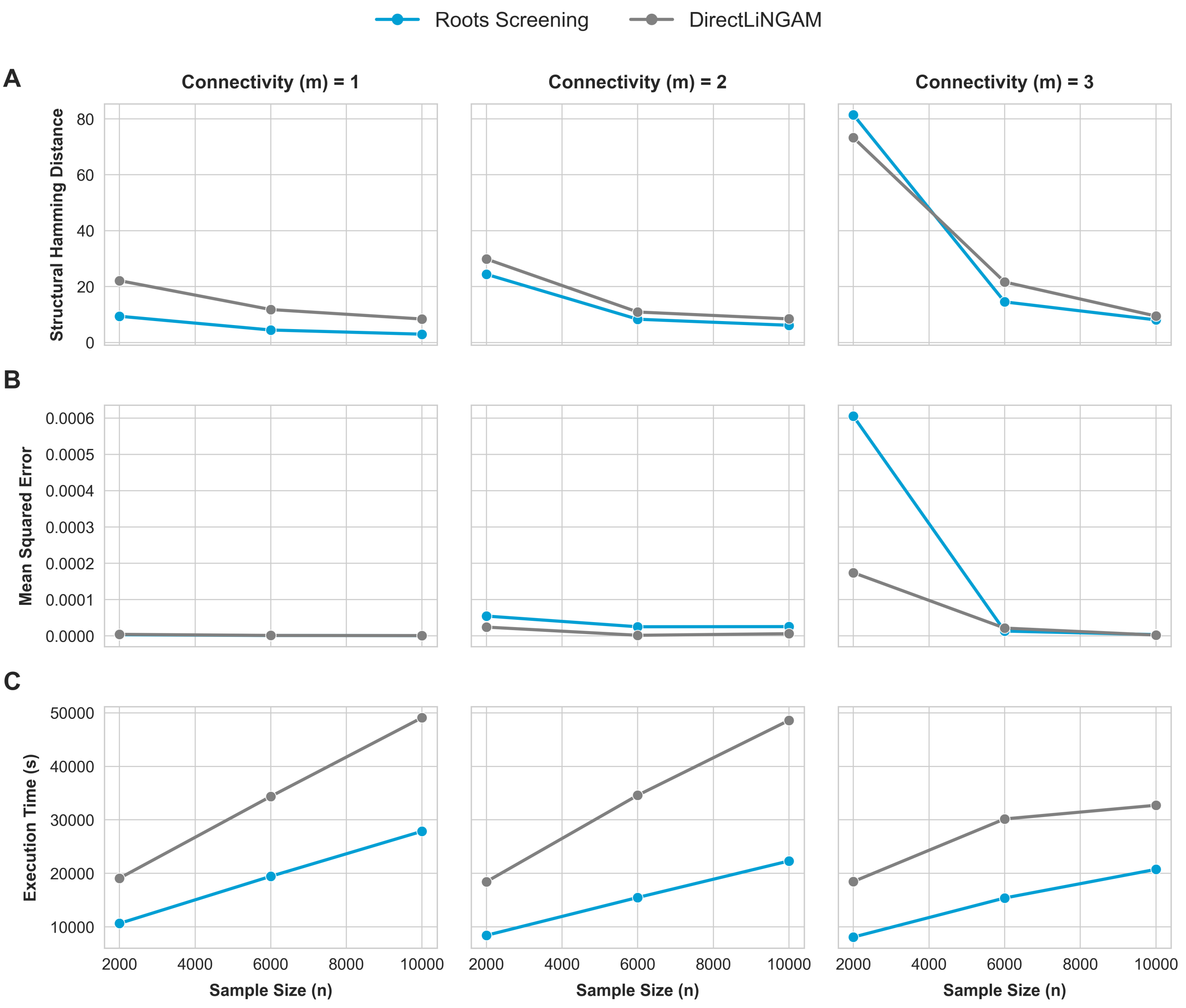

Figure S4. Structural error, estimation error, and computational efficiency across varying sample sizes (n) and connectivity regimes (m) averaged over 20 independent runs. Panels display **(A)** Structural Hamming Distance (SHD), **(B)** Mean Squared Error (MSE), and **(C)** Execution Time in seconds. Roots Screening consistently achieves lower or near the same structural and parametric error across all connectivity settings while maintaining a highly competitive computational execution time.

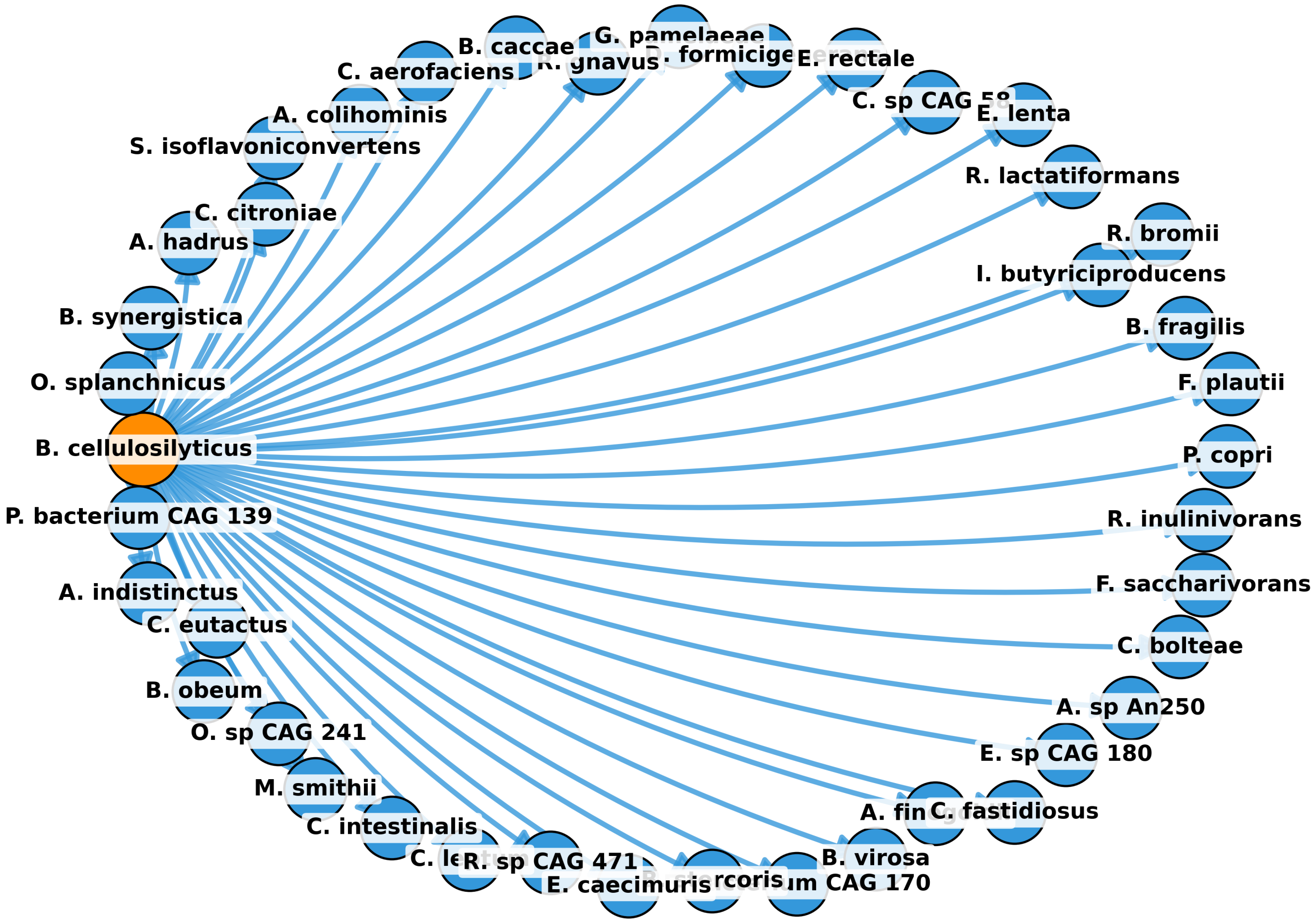

Figure S5. Detailed local causal neighborhood for the root microbial driver *Bacteroides cellulosilyticus*. The panel presents the direct causal downstream footprint originating from this specific taxon. The orange node represents the primary root driver (zero in-degree), while blue nodes denote downstream responder taxa. Node sizes are proportional to their global out-degree.

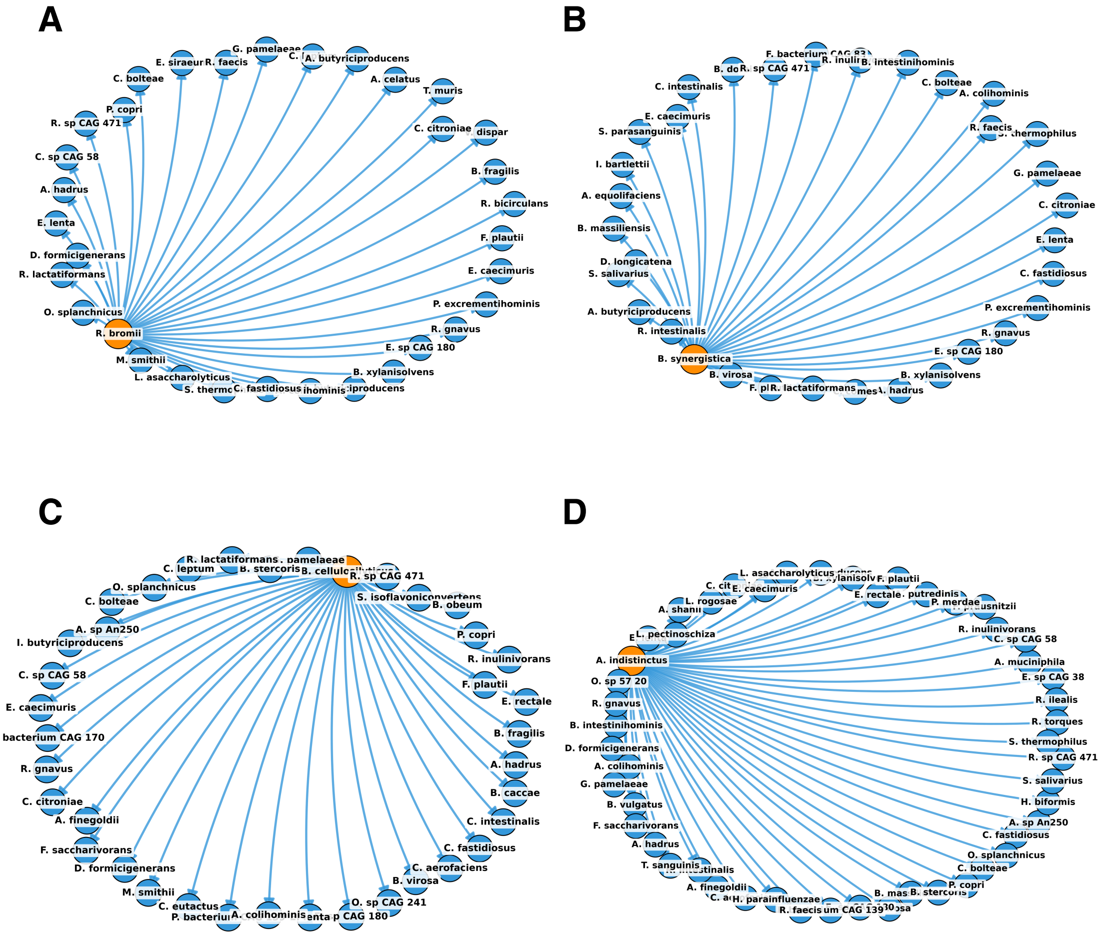

Figure S6. Detailed local causal neighborhoods for the four primary root microbial drivers identified by the Roots Screening procedure. Panels present the direct causal downstream footprints for: **(A)** *Ruminococcus bromii* , **(B)** *Butyricimonas synergistica* , **(C)** *Bacteroides cellulosilyticus*, and **(D)** *Alistipes indistinctus*. Orange nodes represent the primary root drivers (zero in-degree). Node sizes are proportional to their global out-degree.

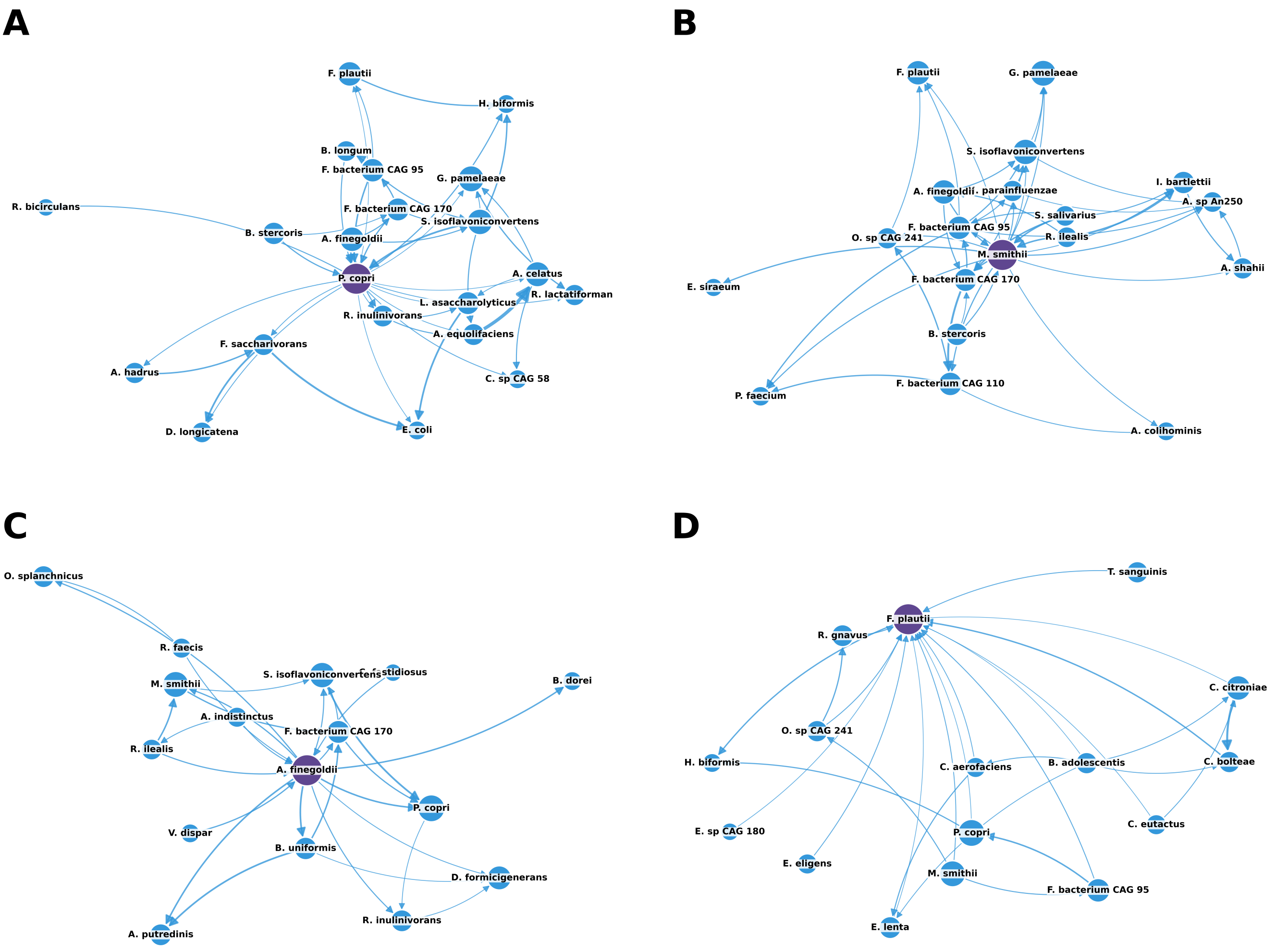

Figure S7. Detailed local causal neighborhoods extracted from the network estimated via eBIC DirectLiNGAM. Panels present the direct causal relationships for selected taxa: **(A)** *Prevotella copri*, **(B)** *Methanobrevibacter smithii*, **(C)** *Alistipes finegoldii*, and **(D)** *Flavonifractor plautii*. The purple node highlights the target taxon, while blue nodes represent the connected neighborhood taxa. Node sizes reflect their global degree within the global network.

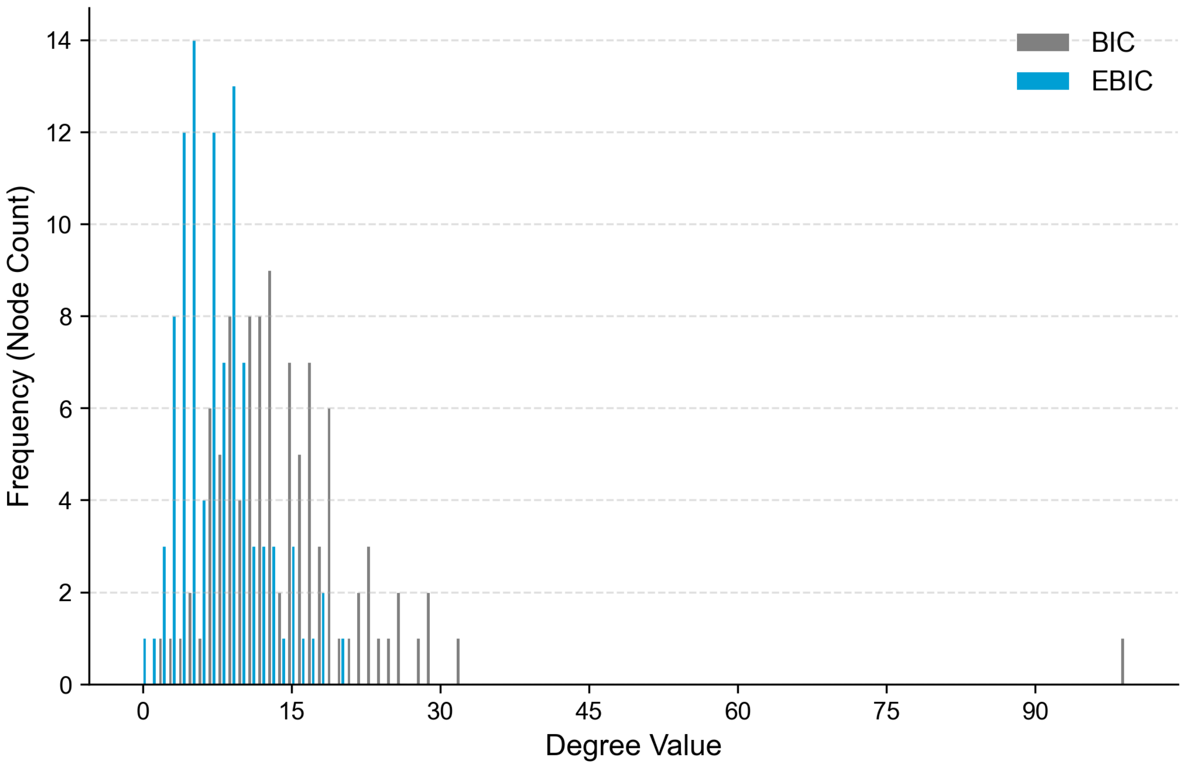

Figure S8. Total degree distribution of the estimated global causal network. The bar chart displays the combined number of causal connections (in-degree plus out-degree) for the eBIC and the BIC approach.
